# Mobile DNA transposition in somatic cells

**DOI:** 10.1186/1741-7007-9-62

**Published:** 2011-09-29

**Authors:** Haig H Kazazian

**Affiliations:** 1 Institute for Genetic Medicine, Johns Hopkins University School of Medicine, Baltimore, MD 21205, USA

## Abstract

It had been long assumed that almost all insertions of mobile DNA elements occurred during germ-cell development rather than in somatic-cell development, but solid evidence for transposition in somatic cells is now accumulating. To add to this evidence, a recent paper in *Mobile DNA *reports the somatic transposition of a site-specific retrotransposon, R2, into its insertion site in 28S ribosomal DNA in *Drosophila *embryos.

See research article: http://www.mobilednajournal.com/content/2/1/11

## 

Mobile DNA, or transposable elements, comprises much of the genome in all organisms, and constitutes around 50% of the genomes of most plants and mammals. Some transposable elements, known as DNA transposons, move by a simple 'cut-and-paste' mechanism removing DNA from one site and inserting it into a new target site. Others, called retrotransposons, move via an RNA intermediate that is copied into DNA and integrated into the genome. Retrotransposons that contain major activities necessary for their mobility are called autonomous. They encode reverse transcriptase and endonuclease activities essential for transposition: other activities that may be required are provided by the host cell. Non-autonomous retrotransposons lack genes for reverse transcriptase and endonuclease and can only move if these activities are provided by an autonomous mobile DNA in the same cell (Figure 1).

**Figure 1:**
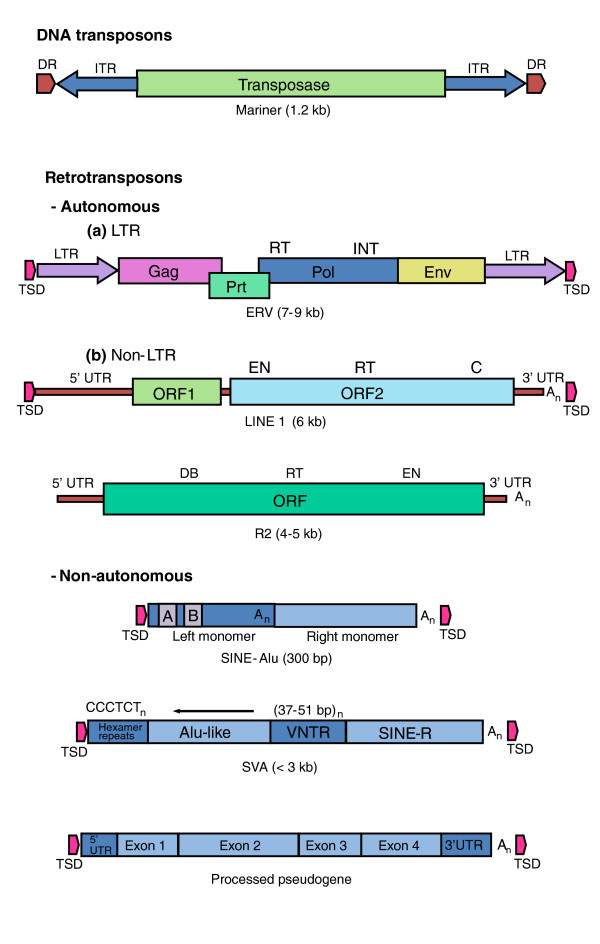
**Types of transposable element**. DNA transposons such as the Mariner-like elements in mammalian genomes are inactive relics of mobile DNAs that transpose directly. They encode a transposase activity that mediates their excision from, and integration into, DNA. Retrotransposons transpose via an RNA intermediate that is converted by a reverse transcriptase (RT) into a DNA that is integrated into the host-cell genome. Retrotransposons that contain many (but not all) of the activities necessary for their mobility are called autonomous. They comprise the endogenous retroviruses (ERVs), which are characterized by **(a) **long terminal repeats (LTRs) and **(b) **the LINEs, which are non-LTR retrotransposons. Of the LINES, only the L1 group is known to be actively mobile in eutherian mammals. R2 is a site-specific element that is active in insects and is the element studied by Eickbush and Eickbush [5]. Non-autonomous elements, such as Alu and SVAs, are dependent on L1 for their mobility. Processed pseudogenes derive from spliced RNAs that are copied and inserted in the genome by the L1s. Mariner: DR, direct repeat (in the host-cell DNA); ITR, inverted terminal repeat. ERV: TSD, target-site duplication (in the host cell DNA); LTR, long terminal repeat; Pol, RNA-dependent DNA polymerase with a reverse transcriptase (RT) domain and an integrase (INT) domain; Env, Gag and Prt encode other proteins required for the virus life cycle. LINE: EN, endonuclease domain; RT, reverse transcriptase domain; C, zinc knuckle domain; DB, DNA binding; A_n_, poly(A). SINE: A/B, A- and B-box PolIII promoter. Reproduced with permission from [1].

The only transposable elements that are autonomous and active in humans and other primates are the LINE-1 (L1) elements [1], which are a type of retrotransposon that lacks the long terminal repeats (LTRs) typical of endogenous retroviruses. L1 elements can also drive the insertion of non-autonomous retrotransposons, which in mammals include Alu, the SVA elements, and processed pseudogenes - the last of which are spliced RNAs that are copied into DNA and inserted in the genome by L1 activities (Figure 1).

A major question in retrotransposon biology is: when does most retrotransposition occur? Because these DNA elements are scattered throughout genomes and inherited from one generation to the next, the answer for many years has been germ cells, as most somatic insertions would not be heritable in the next generation and would not show up in the genome over generations. However, a growing body of evidence has indicated that somatic retrotransposition in mammals not only occurs, but is likely to occur at a substantial frequency.

## R2 retrotransposition in fruit flies

Evidence for the somatic transposition of mobile DNAs in life forms other than mammals is mixed. Tc1, the major DNA transposon of *Caenorhabditis elegans*, is able to transpose in somatic cells [2], whereas in *Drosophila *species, P elements and the I factor, an L1-like non-LTR retrotransposon, appear limited to germ-cell mobility [3,4]. Now, Eickbush and Eickbush, in a study published in *Mobile DNA *[5], find that transposition of another *Drosophila *autonomous retrotransposon, R2, can occur in somatic cells during early embryonic development. R2 is a non-LTR retrotransposon with many features in common with mammalian L1 elements, most notably its ability to reverse transcribe and integrate in a single step directly into chromosomal DNA [6]. However, R2 differs from L1 in that it inserts at a single site in the 28S rRNA genes whereas L1 can enter the genome at a very large number of essentially random, short consensus sequences. In addition, the endonucleases encoded by the two elements differ in their position within the element and their enzymatic type (restriction enzyme type IIs for R2 and apurinic/apyrimidinic endonuclease for L1).

Using single-step PCR, Eickbush and Eickbush found evidence for 15 somatic early embryonic insertions in 7 of 29 flies studied. This number of somatic insertions is clearly a minimum, and one wonders how many more insertions would have been detected with two-step, nested PCR. The detected insertions were present in multiple tissues of both the adult and larval stages, and had all the characteristics of authentic R2 insertions. They occurred at, or very close to, the 28S rRNA gene insertion site of R2. Many had a few non-templated nucleotides at the 5' end, and all were 5' truncated. Because the same somatic insertions were found in different tissues, the timing of many events could be estimated as early in development, before the differentiation of tissues, including the germ line.

A previous study of 213 R2 insertions in the offspring of a single female fly found 32 new insertions [7]. Twenty-seven of these were clear germline events, occurring in a single fly each. The remaining five appeared to be derived from somatic events because the identical insertion occurred in more than one fly, meaning that one parent was a germline mosaic and had the same insertion present in many but not all germ cells. It was also previously known that R2 retrotransposition occurs much less frequently in males than in females, and now Eickbush and Eickbush suggest that perhaps all of male R2 retrotransposition may be due to germline mosaicism. Thus, it appears that the incidence of somatic R2 retrotransposition is not very dissimilar from that of germline R2 insertion.

## Somatic L1 insertions in humans and mice

Over the past six years substantial evidence for somatic insertion of mammalian L1 elements has accumulated. Muotri *et al. *[8] found L1 retrotransposition in neuronal precursors in mouse brains, specifically hippocampus, using engineered human L1 transgenes. Coufal *et al. *[9] used quantitative PCR to expand those data to include increased endogenous L1 insertions in mouse and human brain regions compared to other tissues. Muotri *et al. *[10] subsequently showed that mice with a knockout for the gene *MeCP2*, encoding the methyl-CpG-binding protein 2, have more engineered L1 insertions from L1 transgenes in the hippocampus compared with normal mice. They also found that female human patients with the neurodevelopmental disorder Rett syndrome (who have a natural deficiency of MeCP2) have small but significant increases of L1 insertions in their hippocampi. Meanwhile, van den Hurk *et al. *[11] had demonstrated an example of early embryonic L1 insertion in a human who exhibited both germline and somatic mosaicism for an L1 insertion, Garcia-Perez *et al. *[12] showed retrotransposition of a transfected L1 in human embryonic stem cells, and Kano *et al. *[13] showed that human and mouse L1 transgenes produce more retrotransposition events in early embryogenesis in mice and rats than in the germline. Very recently, Baillie *et al. *[14] have used enrichment of human retrotransposons followed by next-generation sequencing to discover an amazing number (thousands) of somatic retrotransposition events in the hippocampus. Thus, there is no longer any doubt that somatic insertion of L1 occurs in humans and mice.

These findings beg many questions. Transposition of mobile DNA can be destructive to gene function and host organisms possess mechanisms for suppressing transposition, especially in the germline. So why does somatic retrotransposition occur? Why don't host controls on retrotransposition block it in early embryogenesis? From the point of view of the evolution of the animal host, if retrotransposition is likely to occur at all, it is least damaging if it occurs in somatic cells, which are not heritable in the next generation, rather than in the germline, where it may cause mutations that can be passed on to offspring. In addition, somatic insertions are less likely to be immediately detrimental to the host than germline insertions as they affect only a limited number of cells. Thus, if host resources to control retrotransposition are limited, they would best be used in the developing germline.

In the future, we need to learn why various hosts allow somatic insertions for one element but not for another, and what is the frequency of somatic retrotransposition of various mobile DNAs in a variety of organisms and tissue types. We should also learn more about the stages of embryonic development in which most somatic insertions occur, and the role of mobile DNAs, if any, in oncogenesis. Perhaps we will also discover whether controls on transposon mobility in somatic cells in various hosts are similar to those used to control mobility in the germline. In any case, the study by Eickbush and Eickbush [5] provides new evidence for the importance of somatic insertion of L1-like non-LTR retrotransposons.
